# Transcriptional profiles underlying parent-of-origin effects in seeds of *Arabidopsis thaliana*

**DOI:** 10.1186/1471-2229-10-72

**Published:** 2010-04-20

**Authors:** Sushma Tiwari, Melissa Spielman, Reiner Schulz, Rebecca J Oakey, Gavin Kelsey, Andres Salazar, Ke Zhang, Roger Pennell, Rod J Scott

**Affiliations:** 1 Department of Biology and Biochemistry, University of Bath, Claverton Down, Bath BA2 7AY, UK; 2 Department of Medical and Molecular Genetics, King's College London School of Medicine at Guy's, King's College and St Thomas' Hospitals, 8th Floor Guy's Tower, London SE1 9RT, UK; 3 Laboratory of Developmental Genetics and Imprinting, The Babraham Institute, Babraham Research Campus, Cambridge CB2 4AT, UK; 4 Ceres Inc., 1535 Rancho Conejo Boulevard, Thousand Oaks, CA 91320, USA

## Abstract

**Background:**

Crossing plants of the same species but different ploidies can have dramatic effects on seed growth, but little is known about the alterations to transcriptional programmes responsible for this. Parental genomic imbalance particularly affects proliferation of the endosperm, with an increased ratio of paternally to maternally contributed genomes ('paternal excess') associated with overproliferation, while maternal excess inhibits endosperm growth. One interpretation is that interploidy crosses disrupt the balance in the seed of active copies of parentally imprinted genes. This is supported by the observation that mutations in imprinted FIS-class genes of *Arabidopsis thaliana *share many features of the paternal excess phenotype. Here we investigated gene expression underlying parent-of-origin effects in *Arabidopsis *through transcriptional profiling of siliques generated by interploidy crosses and FIS-class mutants.

**Results:**

We found that fertilized *fis1 *mutant seeds have similar profiles to seeds with paternal excess, showing that the shared phenotypes are underpinned by similar patterns of gene expression. We identified genes strongly associated with enhanced or inhibited seed growth; this provided many candidates for further investigation including MADS-box transcription factors, cell cycle genes, and genes involved in hormone pathways.

**Conclusions:**

The work presented here is a step towards understanding the effects on seed development of the related phenomena of parental genome balance and imprinting.

## Background

Crossing plants of the same species but different ploidies often alters seed development, generating reciprocal phenotypes depending on the direction of the cross [1]. Particularly dramatic effects are seen in endosperm, a fertilization product derived from the diploid central cell and a haploid sperm that transfers nutrients from the seed parent to the developing or germinating embryo. In general, an increased ratio of paternally to maternally contributed genomes in the seed ('paternal excess')--generated for example by crossing a diploid seed parent with a tetraploid pollen parent--is associated with increased growth of endosperm, while an increased ratio of maternal to paternal genomes ('maternal excess') inhibits endosperm growth. A widely accepted interpretation of interploidy cross phenotypes is that they disrupt the balance in the seed of active copies of parentally imprinted genes, which, depending on the particular gene, are expressed from only the maternal or only the paternal alleles [1,2]. Study of imprinting in plants has focused on two model species, *Arabidopsis thaliana *and *Zea mays*; in both, parent-specific expression of imprinted genes is largely or exclusively confined to endosperm, with expression generally either biallelic or absent in the embryo [3-12]. To date, *MATERNALLY EXPRESSED IN EMBRYO *(MEE1) is the only known gene that is monoallelically expressed in the embryo [13].

In *Arabidopsis*, crosses between diploid and tetraploid plants often produce viable triploid embryos, with 2xX4x crosses (diploid seed parent and tetraploid pollen parent) generating heavy seeds containing large embryos, and 4xX2x crosses producing light seeds with small embryos [14]. Paternal excess is associated with increased and prolonged proliferation of the endosperm, overgrowth of the chalazal endosperm and associated nodules, an abnormally large seed cavity, and delay in endosperm cellularization (normal endosperm development in *Arabidopsis *is described by Olsen, [15]), while maternal excess is characterized by inhibited endosperm division, small chalazal endosperm, absence of nodules, small seed cavity, and precocious cellularization and mitotic arrest of endosperm. Therefore the extent of endosperm growth is an important component in the final size of the seed and embryo even in a species with only one cell layer of endosperm remaining in the mature seed. Reciprocal crosses between diploid and hexaploid Arabidopsis plants produce more extreme versions of these phenotypes, and this level of parental genomic imbalance is also lethal: embryos arrest by heart stage, and the seeds abort. In maize, crosses between diploid and tetraploid plants usually result in seed abortion [16], but before this occurs, the developing seeds also display reciprocal phenotypes, with delayed endoreduplication (i.e. delayed differentiation) observed in kernels with paternal excess, and early mitotic arrest when there is maternal excess [17].

In *Arabidopsis*, a maternal mutation in any one of four 'FIS-class' genes--*FERTILIZATION INDEPENDENT SEED 1 *(*FIS1*)/*MEDEA *(*MEA*), *FIS2*, *FIS3*/*FERTILIZATION INDEPENDENT ENDOSPERM *(*FIE*), or *MULTICOPY SUPPRESSOR OF IRA 1 *(*MSI1*)--leads to seed abortion, with phenotypes including overexpansion of the seed cavity, failure of endosperm cellularization, large chalazal endosperm and nodules, and arrest of embryos around heart stage [18-22]. Intriguingly, these phenotypes are very similar to those resulting from interploidy crosses that generate a lethal level of paternal excess [14,19,23]. *msi1 *mutants deviate somewhat from this model in producing fewer peripheral endosperm nuclei than wild-type seeds, although chalazal endosperm and nodules are still enlarged [22]. *FIS1/MEA *was the first imprinted gene to be described in *Arabidopsis*. In endosperm, *FIS1/MEA *is expressed only from the maternally derived alleles, while it is biallelic in embryo, at least from torpedo stage onward [3,5]. *FIS2 *is expressed only from maternal alleles in developing seeds [11]. There is no molecular evidence for imprinting of the other FIS-class genes. The four FIS-class gene products participate in Polycomb Repressive Complex (PRC) 2 [21,24,25], which, like animal PRCs inhibits transcription of target genes through epigenetic modification of chromatin [26,27]. The similarity of FIS-class mutant and lethal paternal excess phenotypes led to the proposal that one function of the wild-type FIS proteins is to repress transcription of loci in the maternally derived genome that are normally expressed only when paternally contributed [23]. This has been supported by the discovery that the *FIS1/MEA-*containing PRC2 represses the maternal alleles of *PHERES1 *(*PHE1*), an imprinted gene which is preferentially expressed from the paternal genome [10,28].

Another feature of FIS-class mutants is an 'autonomous endosperm' phenotype. Wild-type *Arabidopsis *embryo sacs must be fertilized before the egg or central cell will divide. In contrast, if FIS-class mutant flowers are prevented from self-pollinating, the central cell nevertheless divides and forms a syncytial structure resembling peripheral endosperm, even though there is no cellularization or regional specification [19,21,29,30]. In *msi1 *mutants, the unfertilized egg also divides to form a small multicellular structure expressing several markers for embryogenesis [31]. The seed-like structures produced by unfertilized FIS-class mutant ovules invariably abort. Comparison of gene expression in fertilized and unfertilized FIS-class mutants could reveal genes that are normally repressed by PRC2 in seeds.

In the work presented below, we investigated gene expression underlying paternal and maternal excess phenotypes using two different microarray platforms, and also profiled fertilized and unfertilized FIS-class mutants. This project had several goals, including the identification of genes that are not necessarily imprinted, but which are nevertheless associated with promoting or inhibiting seed growth; and comparing gene expression between seeds from interploidy crosses and FIS-class mutants. Another goal was to gather molecular data to help us position fertilized and unfertilized FIS-class mutants on the maternal-paternal spectrum. We found that fertilized *fis1 *mutant seeds have similar transcriptional profiles to seeds with paternal excess, showing that the shared phenotypes are underpinned by similar patterns of gene expression. To learn more about regulation of seed size, we filtered our data for sets of genes strongly associated with enhanced or inhibited seed growth. Our results illustrate the molecular link between paternal excess and FIS-class mutations, and potentially provide tools for altering seed size.

## Results and Discussion

### Generation of samples and hybridization to arrays

To explore the patterns of gene expression underlying the phenotypes of seeds generated by interploidy crosses and FIS-class mutants, we performed two independent microarray experiments using biological replicate samples and different array platforms to increase confidence in the results. Other cross-platform comparisons have been successful in *Arabidopsis *[32].

For our first experiment, RNA was extracted from siliques at 5 DAP resulting from the crosses 6xX2x and 4xX2x (maternal excess), 2xX2x (balanced), 2xX4x and 2xX6x (paternal excess), and *fis1/mea*X2x, and hybridized to custom Agilent 22K two-dye (Cy3 and Cy5) arrays (http://www.chem.agilent.com; http://www.ceres-inc.com). For the second experiment, RNA was extracted at 5 DAP from two further independent biological samples of the crosses listed above, and also from unfertilized siliques of male-sterile *msi1 *mutants at 7 days after floral opening (see Methods), and hybridized to Affymetrix ATH1 full-genome chips http://www.affymetrix.com. Thus, our experiments incorporated seeds from interploidy crosses generating both viable and lethal parental imbalance, a fertilized FIS-class mutant that develops with a phenotype resembling lethal paternal excess, and an unfertilized FIS-class mutant that develops with no paternal contribution.

**Figure 1:**
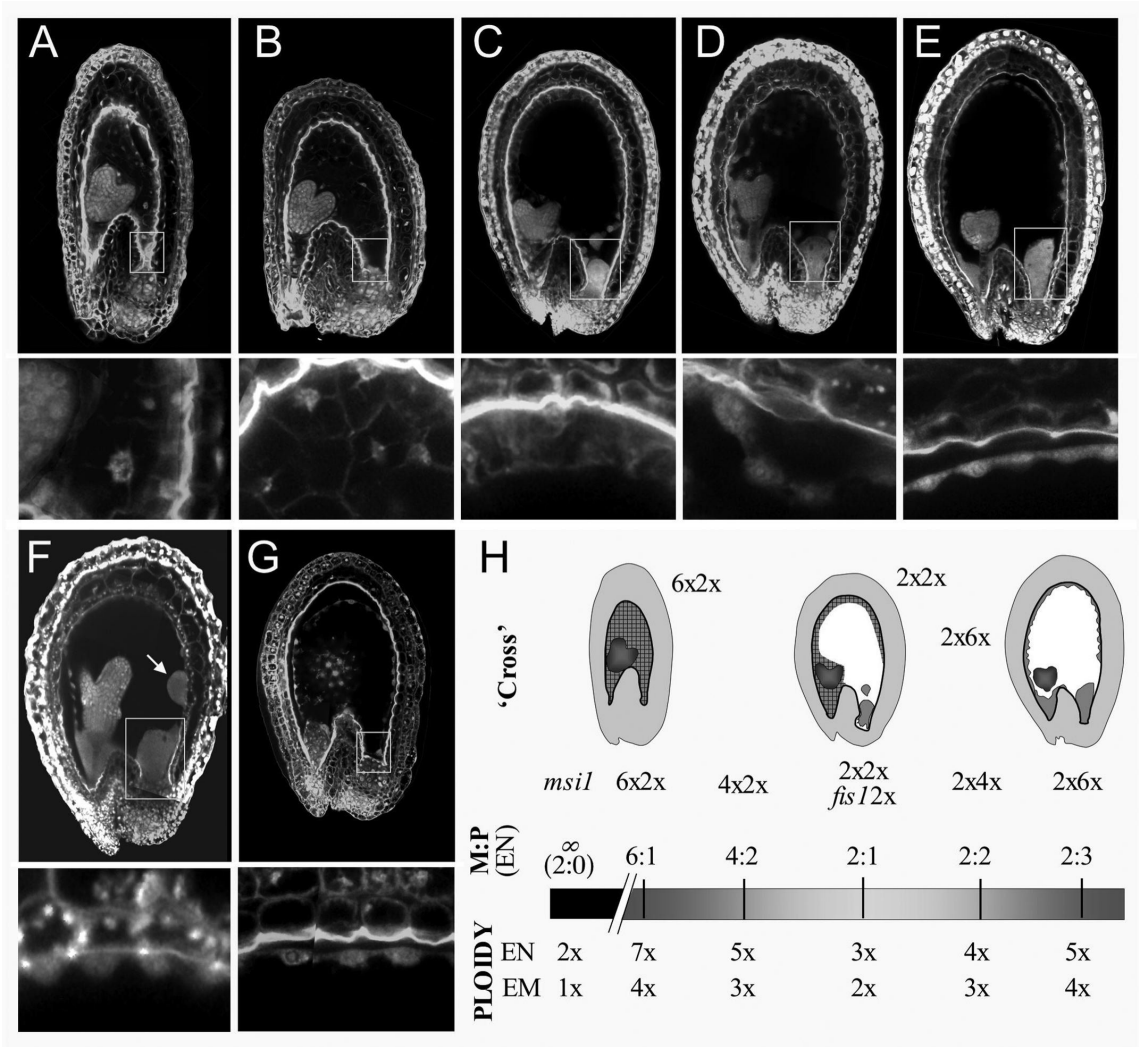
**Phenotypes and genetic constitution of seeds used for transcriptional profiling**. **(A-G) **Confocal micrographs of seeds at 5 days after pollination (7 days after flowering for *msi1*). Boxes surround the chalazal region. Below each composite seed image is an enlargement showing peripheral endosperm. Seed genotypes are: **(A) **6xX2x, **(B) **4xX2x, **(C) **2xX2x, **(D) **2xX4x, **(E) **2xX6x, **(F) ***fis1*X2x, **(G) ***msi1 *(autonomous seed produced by an unfertilized *msi1*mutant ovule). Arrow in **(F) **indicates endosperm nodules. **(H) **Spectrum of maternal:paternal genomic balance. Above bar: ratio of maternally to paternally derived genomes in the endosperm for each 'cross' (sexual crosses + unfertilized *msi1*). Below bar: absolute number of genomes in endosperm (EN) and embryo (EM) for each 'cross'.

The phenotypes of all crosses are illustrated in Figure 1. At 5 DAP, seeds with the normal balance of maternal to paternal genomes (2xX2x cross, generating a 2m:1p endosperm) typically contain a heart-stage embryo, peripheral endosperm which has begun to cellularize from the micropylar pole, a compact chalazal endosperm, and endosperm nodules (Fig. 1C). In seeds with paternal excess (2xX4x and 2xX6x) there is no cellular endosperm at this stage, and the chalazal endosperm is enlarged (Fig. 1D, E). *fis1*X2x seeds (Fig. 1F) likewise contain only free-nuclear endosperm, and in common with 2xX6x crosses, the endosperm never cellularizes and the seeds abort [14,18,33]. Fertilized *fis1 *mutants also produce greatly enlarged endosperm nodules. Therefore the characteristic phenotypes both of paternal excess and of a fertilized FIS-class mutant include overproliferation of endosperm and delay or failure of cellularization. At the other end of the phenotypic spectrum, seeds with maternal excess (4xX2x and 6xX2x) produce small endosperms that cellularize precociously, and tiny chalazal endosperms with no associated nodules (Fig. 1B, A). The endosperm fails to cellularize in unfertilized *msi1 *mutants (Fig. 1G) but these also lack chalazal endosperm, so are difficult to classify according to endosperm growth and morphology.

Figure 1H illustrates the absolute numbers of genomes in embryo and endosperm for each seed genotype, and places each seed on a spectrum of maternal:paternal genomic balance in the endosperm. Although *fis1*X2x seeds contain the same number of genomes and the same genomic balance as 2xX2x, both phenotypic (endosperm overproliferation) and molecular evidence (ectopic expression of the normally repressed maternal allele of *PHE1*) indicates that they may be regarded as having 'virtual' paternal excess [10,23]. Unfertilized *msi1*mutants contain no paternal genomes, but may also share some features of paternalization due to the mutation.

RNA hybridization and statistical analysis for each microarray platform are described in the Methods. Results for the Affymetrix and Agilent experiments are available through GEO http://www.ncbi.nlm.nih.gov/geo under the accession number GSE20007. Correspondence between Affymetrix and Agilent probes was determined as described in the Methods. This yielded a list of 20,442 genes that we used in the subsequent analysis (cf. 33,518 Arabidopsis genes annotated in TAIR Release 9.0 (http://www.arabidopsis.org, [34]). We subtracted from this list all genes that were below a threshold of absolute expression in all samples, or which had widely differing ratios in the dye-swap experiments indicating unreliable expression (see Methods), giving a final list of 14,944 unique AtIds that were called present in at least one sample. These AtIds, along with the corresponding Agilent and Affymetrix probesets and averaged SLR and pSLR values (see Methods), are provided in Additional file 1 table S1 online. Interestingly 1,947 of the 2,608 genes predicted to be early seed specific by Day et al [35], were found to be present in our dataset (Additional file 1 table S1.1.). Of the genes called present in the peripheral endosperm of the Goldberg and Harada dataset http://seedgenenetwork.net/arabidopsis a similar proportion (74%) were also represented in our dataset (data not shown). This gives us the confidence that using whole siliques as the experimental material captures a significant proportion of the endosperm expressed genes.

### Differential transcript accumulation reflects altered developmental programmes, not increased gene dosage

To identify genes that were over- or underexpressed ('up' or 'down') in each interploidy cross or FIS-class mutant relative to 2xX2x, we generated lists of genes with signal-log ratios (SLRs) ≥ 0.6 or ≤ -0.6, corresponding to changes in expression of approximately 50% up or down; many genes had much higher changes than this. For Affymetrix data we based the lists on p-value weighted SLRs (pSLR) to minimize interference from values for which there was little statistical evidence for differential expression (see Methods and [36]). Genes called up or down together with their SLRs/pSLRs are presented in Additional file 2 table S2 online. We next compared over- and underexpressed genes across platforms for each cross; for example we ultimately only called a gene up if its SLR was ≥ 0.6 in both Affymetrix and Agilent datasets. The lists of genes called up and down that were supported by both platforms are provided in Additional file 3 table S3 online.

Table 1 shows the numbers of genes called up or down in each sample and platform, along with genes in common across platforms. 2xX4x crosses showed the least amount of misexpression, and 6xX2x the greatest. In *fis1*X2x seeds, which differ from wild-type by a premature stop codon in a single ORF [18], 401 genes were called up in both platforms, while in phenotypically similar 2xX6x crosses, which have triple the normal number of paternal genomes in the seed, only 288 genes were called up. This suggests that interploidy crosses provide less disturbance to gene expression than FIS-class mutations and can be a valid route to exploring imprinting. We also observed that in the interploidy crosses there was no general bias towards overexpression of genes, though the seeds all contained more genomes than 2xX2x seeds (Fig. 1). This rules out a simple relationship between gene dosage and level of gene expression, instead indicating that differential gene expression observed here in many cases represents genuine changes to developmental programmes.

**Table 1:** Cross-platform comparison of genes called up and down.

Cross	Affymetrix	Agilent	overlap	% agreement	% transcription factors
**2x X 4x up**	597	755	157	26.3	12.1
**2x X 4x down**	818	378	93	24.6	14
**2x X 6x up**	821	814	288	35.4	10.8
**2x X 6x down**	877	1072	392	44.7	13.6
**4x X 2x up**	1164	870	300	34.5	7.3
**4x X 2x down**	1854	801	326	40.7	8.6
**6x X 2x up**	1388	2109	776	55.9	9.7
**6x X 2x down**	1859	2333	921	49.5	7.5
**fis1 X 2x up**	1068	2582	401	37.5	10.5
**fis1 X 2x down**	1904	3039	710	37.3	11.3
**msi1 up**	1691				10.2
**msi1 down**	2414				7.6

The numbers of genes called up or down for each cross and each platform are shown, along with the numbers of genes in common across both platforms for each cross ('overlap'), and the percentage of the maximum possible agreement between platforms. The right-hand column shows the percentage of each list consisting of transcription factors (http://datf.cbi.pku.edu.cn; Guo et al., 2005). For all samples except for *msi1 *the percentage of transcription factors was calculated from the lists of genes called up or down in both platforms ('overlap'). See Additional file 2 table S2 for genes called up or down in each platform and Additional file 3 table S3 for genes called up or down in both platforms.

The extent of agreement between the Affymetrix and Agilent datasets was calculated as the percentage of genes in common out of the possible maximum (Table 1). The overlap between datasets ranged between 24.6 and 55.9%; this compares favourably with the results of Pylatuik and Fobert [32], who recorded cross-platform overlaps of 14.3 to 25.5% between sets of Arabidopsis genes overexpressed 2.5-fold.

**Figure 2:**
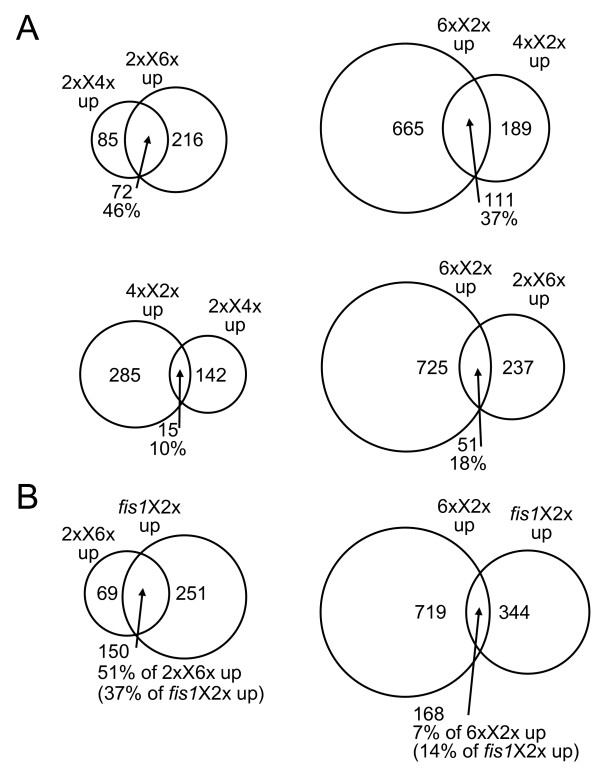
**Extent of agreement between sets of genes called up**. The percentage shown in the region of overlap is the percentage of the maximum possible agreement for each pair. **(A) **Agreement between interploidy crosses generating paternal and maternal excess. **(B) **Agreement between *fis1*X2x and the two extreme interploidy crosses.

We next investigated whether the sets of genes called up in the two crosses generating paternal excess had more in common with each other than with genes up in maternal excess, and vice versa. The results of these comparisons are represented in Figure 2. We found that datasets from phenotypically similar crosses had a higher proportion of genes in common than datasets from opposite crosses. For example, the correspondence between genes called up in 2xX4x and 2xX6x was 46% of the possible maximum (the number of genes in the smaller set, in this case 2xX4x up), although one of these crosses produces viable seeds and the other is lethal; while the overlap between genes up in 2xX4x and 4xX2x seeds, which are both viable, was only 10% of the maximum. These results also indicate that overexpression observed in these experiments is much more likely to reflect genuine changes in developmental programmes than simply the presence of extra copies of genes in seeds with increased ploidy. We next compared genes called up in *fis1*X2x with those up in the two lethal interploidy crosses. More than 50% of genes overexpressed in 2xX6x were also up in *fis1*X2x, while only 7% of genes up in 6xX2x were up in *fis1*X2x, supporting the hypothesis that FIS-class mutations have a paternalizing effect on seed development.

We assayed gene expression at a relatively early stage since we are especially interested in identifying genes that control seed development. Nevertheless, we expected that some genes would be over- or underexpressed as a consequence of altered development rather than as a cause. To assess whether genes showing differential expression at 5 DAP could be regulators of seed development, we compared the list of 1922 transcription factors annotated in the Arabidopsis genome (http://datf.cbi.pku.edu.cn; [37]) with the sets of genes we called up or down. There are 27,379 protein coding genes annotated in TAIR release 9.0 http://www.arabidopsis.org. So, approximately 7.0% of the genome consists of transcription factors. A similar proportion, 8% (1,192), of the genes called present in our experiment are on the transcription factor list. However most of our up or down lists contained a higher proportion than this (Table 1). We conclude that genes controlling seed development, rather than merely responding to upstream changes, are well represented in our transcription profiles.

### Validation of microarray data

To validate the microarray data we examined the expression of 20 genes using quantitative real-time PCR (qRT-PCR). These genes were mainly chosen because of their expression trends in the microarray data, and some will be discussed in more detail below. Results are presented in Additional file 4 table S4 online and summarized in Table 2, which shows the extent of agreement between each microarray platform and qRT-PCR for the 20 genes. Out of 220 calls tested (11 calls for each of 20 genes), microarray and qRT-PCR data gave the same call for 170 (i.e. for a particular cross and platform, a gene was called up, down, or unchanged in both the microarray and the qRT-PCR experiments), giving overall agreement of 77%. A similar level of agreement between microarray and qRT-PCR data for transcription in maize anthers was recently reported by Skibbe et al. [38]. Where our microarray and qRT-PCR calls did not agree, the majority nevertheless had fold changes in the same direction (Additional file 4 table S4). Only one sample, 4xX2x Agilent, had less than 65% agreement with the qRT-PCR findings. None of the genes from this dataset where qRT-PCR and the Agilent data disagreed were called changed in both Agilent and Affymetrix platforms, and therefore were not included in our final list of genes called down in this cross. We conclude from our validation that the microarray data is particularly robust for genes that show the same expression trend in both platforms.

**Table 2:** Agreement of microarray and qRT-PCR data.

	msi1	6xX2x	4xX2x	2xX4x	2xX6x	fis1X2x
**Affymetrix**	13 (65%)	15 (75%)	18 (90%)	14 (70%)	17 (85%)	18(90%)
**Agilent**		15 (75%)	8 (40%)	17 (85%)	19 (95%)	16 (80%)

Expression of 20 genes was tested using qRT-PCR and calls of up, down, or not changed were compared with calls for the same genes in the microarray data. Each cell shows the number (percentage) of calls that agreed out of a maximum of 20. qRT-PCR results are provided in Additional file 4 table S4.

### Hierarchical clustering of expression data identifies maternal and paternal groups

**Figure 3:**
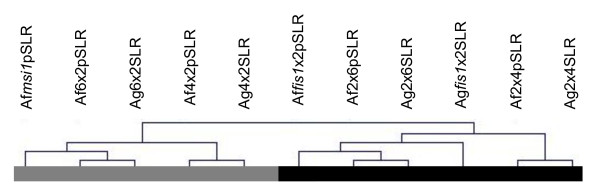
**Hierarchical clustering of gene expression data from two microarray experiments**. The dendrogram indicates the degree of similarity between samples. A black bar marks the paternal cluster and a grey bar the maternal cluster.

We were interested in testing whether the crosses generating paternal or maternal excess had similar expression patterns, and we also wanted to position the fertilized and unfertilized FIS-class mutants on the maternal-paternal spectrum. We used hierarchical clustering [39] to compare expression trends among all samples. Repeated clustering using different distance measures (see Methods) robustly showed distinct 'paternalized' and 'maternalized' transcriptome sets, where 2xX4x, 2xX6x, and *fis*1X2x formed the paternalized cluster and 4xX2x, 6xX2x, and parthenogenetic *msi*1 belonged to the maternalized cluster (Fig. 3). Principal Components Analysis of the microarrays also gave results which are consistent with the hierarchical clustering (not shown).

The transcriptional profile of *fis1*X2x crosses is most similar to the profiles of 2xX6x seeds from both platforms. This supports a model in which FIS-class mutations 'paternalize' the maternally inherited genomes in the endosperm by removing crucial maternal-specific function, so that the balance of maternal:paternal genomes is in effect more like the ratio generated by 2xX6x crosses [23]. Although *msi1 *is also a FIS-class mutant, it was not pollinated for our experiment, and therefore not surprisingly had a very different transcriptional profile from fertilized *fis1 *seeds. *msi1 *clustered most closely with 6xX2x; this may reflect shared features such as near or total absence of chalazal endosperm.

### Association of gene ontologies with different seed phenotypes

To determine whether categories of genes with particular biological function were associated with different seed phenotypes, we looked for GO (gene ontology) biological process terms [40] that were over- or underrepresented among genes showing differential expression, using the GOstat tool available at http://gostat.wehi.edu.au[41]. We compared the list of 14,944 genes called present in the experiment (Additional file 1 table S1) with genes that were called up or down in the interploidy crosses and in *fis1*X2x.

Selected Biological process terms that occurred with significantly enhanced or reduced frequency (P < 0.05, adjusted using the Benjamini and Hochberg correction for multiple testing) are shown in Table 3. Some terms were omitted because of redundancy. Not all up and down lists contained categories with altered frequency. Loci called down in 2xX6x and *fis1*X2x were enriched for genes involved in cell cycle regulation. Downregulated genes in triploid seeds have previously been found to be enriched in genes within the functional category of cell cycle regulation [42]. This was at first surprising given the overproliferation of 2xX6x and *fis1*X2x endosperms, but these seeds also display aberrations in the cell cycle such as failure of endosperm cellularization. Strikingly, 44 of the genes called down in 2xX6x and 22 of the genes called down in fis1X2x are among a set of 82 *Arabidopsis *genes recently identified as showing an expression peak at the G2/M boundary in synchronized cell culture [43]. Mitosis-specific genes called down in 2xX6x and/or *fis1*X2x include the syntaxin *SYP111/KNOLLE *(At1g08560); several kinesins such as *HINKEL/ATNACK1 *(At1g18370) and *ZCF125 *(At1g59540); the mitotic cyclins *CYCB1;4 *(At2g26760), *CYCB1;3 *(At3g11520), and *CYCB1;2 *(At5g06150); *CDKB2;1 *(At1g76540), a cyclin-dependent kinase involved in the G2/M transition; and the myb family transcription factor *MYB3R-4 *(At5g11510). *SYP111/KNOLLE *is expressed in mitotically dividing cells and in cellularizing endosperm, and is required for membrane vesicle fusion in the plane of cell division [44]. Kinesins associate with microtubules and are involved in cytoskeleton organization and transport of vesicles and organelles; HIK/ATNACK1 is essential for cell plate formation during mitotic cytokinesis [45] and ZCF125 is involved in interactions between the spindle and the kinetochores of chromosomes [46]. MYB3R-4 is proposed to regulate genes involved in the G2/M transition [43]. Nearly 80% of the mitosis-specific genes downregulated in 2xX6x are also called down in 6xX2x, perhaps because cell division has ceased in seeds with lethal maternal excess by this stage. Underexpression of genes involved in DNA replication initiation in 6xX2x, is consistent with reduced division in 6xX2x.

**Table 3:** Selected GO biological process terms over- and underrepresented among genes called up and down.

sample	GO term	no. (%) in sample	no. (%) in all genes	P-value bold = underrepresented
all genes in experiment n = 11,091 annotated				
2xX4x down n = 71 annotated	response to stimulus	15(21.12)	817(7.3)	0.00159
	regulation of transcription, DNA-dependent	8(11.26)	374(3.37)	0.0181
				
2xX6x up n = 228 annotated	cell wall organization and biogenesis	8 (3.5)	80 (0.72)	0.0371
	inorganic anion transport	4(1.75)	20(0.18)	0.0483
				
2xX6x down n = 302 annotated	microtubule-based movement	9(2.98)	26(0.23)	2.56 e-06
	regulation of cell cycle	7(2.31)	38(0.34)	0.00426
	steroid metabolic process	4(1.32)	18(0.16)	0.0373
				
fis1X2x up n = 304 annotated	carbohydrate metabolic process	27(8.88)	351(3.16)	7.88 e-06
				
fis1x2x down n = 541 annotated	biosynthetic process	16(2.77)	773(6.96)	0.0342
	electron transport	33(6.09)	352(3.17)	0.0295
	regulation of biological process	63(11.64)	838(7.55)	0.0342
	regulation of cell cycle	8(1.47)	38(0.34)	0.0345
	sequestering of lipid	4(0.73)	8(0.72)	0.0342
	response to chemical stimulus	31(5.73)	348(3.13)	0.0399
				
4x X 2x up n = 219 annotated	cellular process	44(20.09)	3665(33.0)	0.0141
				
6xX2x up n = 554 annotated	defense response	30(5.41)	189(1.70)	8.11 e-09
	response to stimulus	67(12.09)	817(7.36)	0.00345
	response to starvation	5(0.9)	14(0.13)	0.019
	macromolecule metabolic process	73(13.17)	2300(20.74)	0.00246
				
6xX2x down n = 697 annotated	translation	41(5.88)	276(2.49)	2,76 e-06
	macromolecule biosynthetic process	48(6.88)	380(3.42)	5.57 e-05
	macromolecule metabolic process	185(26.54)	2300(20.73)	0.00551
	DNA replication initiation	4(0.57)	7(0.06)	0.0207
	transcription factor import into nucleus	3(0.43)	4(0.04)	0.0367
	DNA metabolic process	29(4.16)	168(1.5)	2.76 e-06
	nucleosome assembly	11(1.57)	28(0.25)	5.57 e-05
	chromatin assembly and disassembly	12(1.72)	38(0.34)	0.00019
	organelle organization and biogenesis	42(6.02)	291(2.62)	2.76 e-06
	cellular component organization and biogenesis	63(9.03)	618(5.57)	0.00282
	microtubule-based movement	7(1.0)	26(0.23)	0.0358

The GOstat tool (http://gostat.wehi.edu.au/; Beissbarth and Speed, 2004) was used to identify GO biological process terms significantly (P < 0.05) over- and underrepresented among genes called up and down. P-values were adjusted using the Benjamini and Hochberg correction for multiple testing. Each list was compared with the set of all genes called present in the experiment.

n = number of genes in each list with GO biological process annotation.

### Association of individual gene expression with seed phenotypes

To learn more about factors involved in controlling seed size, we were interested in identifying the genes most strongly associated with enhanced or inhibited seed growth. To determine the genes with the most robust associations we used several filtering criteria. (1) As described above, we only called a gene up or down in interploidy crosses if this was supported in both platforms. (2) We next looked for intersections of gene sets that were up or down in crosses generating phenotypically similar seeds. For example, to find the genes strongly associated with enhanced endosperm/seed growth we chose genes overexpressed in both 2xX4x and 2xX6x crosses. (3) After identifying genes called up in seeds in one phenotypic class, we applied a further condition that those genes should not be up in the opposite class. This was to avoid false positives from genes that may have been overexpressed because of polyploidy, stress, or other causes affecting seeds irrespective of the extent of their growth. To increase the selectivity of the final lists we used broad definitions for 'not up' and 'not down': we called a gene 'not up' if the Agilent SLR and Affymetrix pSLR were both equal to or below 0.3 (corresponding to a change in expression of approximately 20%), and 'not down' if the SLRs/pSLRs were = -0.3. For example, our list of genes strongly associated with endosperm overproliferation were called 'up' in 2xX4x and 2xX6x, and also 'not up' in 4xX2x and 6xX2x.

The lists of seed class-associated genes, along with annotations and GO biological process terms (http://www.arabidopsis.org; [40]), are presented in Additional file 5 table 5 online. The first group (Additional file 5 table S5, S5.1-3) comprises lists of 'large seed genes' (we have used 'large seeds' as a shorthand for seeds with increased endosperm proliferation, even though in lethal crosses these seeds are only large while they are still alive). These genes are positively associated with large seeds (up in combinations of 2xX4x, 2xX6x, and *fis1*X2x, not up in 4xX2x and 6xX2x), or negatively associated with small seeds (down in combinations of 4xX2x, 6xX2x, and *msi1*, and not down in 2xX4x and 2xX6x). We considered genes up in 2xX4x and 2xX6x and not up in 4xX2x and 6xX2x to be particularly strongly associated with promoting or responding to seed growth; these are shown in Table 4. The second group (Additional file 5 table S5, S5.4-6) contains lists of 'small seed genes', which are positively associated with small seeds (up in combinations of 4xX2x, 6xX2x, and *msi1*, and not up in 2xX4x and 2xX6x) or negatively associated with large seeds (down in 2xX4x or in 2xX6x and *fis1*X2x, not down in 4xX2x and 6xX2x). Genes up in 4xX2x and 6xX2x and not up in 2xX4x and 2xX6x are shown in Table 6. We also constructed a list of genes called up in the two FIS-class mutants (Additional file 5 table S5, S5.7) (up in *fis1*X2x and *msi1*), to identify genes overexpressed in both fertilized and unfertilized seeds with impaired function of the PRC2 that contains FIS1/MEA and MSI1.

**Table 4:** Genes called up in 2xX4x and 2xX6x, not up in 4xX2x and 6xX2x.

AtId	symbol	annotation	GO biological process (function)
At1g03445	BSU1	serine/threonine phosphatase	brassinosteroid mediated signalling
At1g13680		phospholipase C, similar to MAP3K-like protein kinase	intracellular signalling cascade
At1g17770	SUVH7	SU(VAR)3-9 homolog, a SET domain protein	chromatin modification
At1g22090	EMB2204	unknown protein	embryonic development
At1g44090	ATGA20OX5	gibberellin 20-oxidase	(isopenicillin-N synthase activity)
At1g48010		invertase/pectin methylesterase inhibitor family protein	(pectinesterase inhibitor activity)
		small, secreted, cysteine rich protein with sequence similarity to	
At1g60985	SCRL6	SCR (S locus cysteine-rich protein)	signal transduction
At1g65300	PHE2/AGL38	MADS-box family transcription factor	regulation of transcription
At1g65330	PHE1/AGL37	MADS-box family transcription factor	regulation of transcription/embryonic development
		bifunctional cytosolic hydroxymethyldihydropterin	
At1g69190		pyrophosphokinase/dihydropteroate synthase (HPPK/DHPS)	response to oxidative stress
At1g71770	PAB5	polyadenylate-binding protein	translational initiation
		protease inhibitor/seed storage/lipid transfer protein (LTP)	
At1g73560		family protein	lipid transport
At1g73610		GDSL-motif lipase/hydrolase family protein	lipid metabolic process
At1g77510	ATPDIL1-2	protein disulfide isomerase	cell redox homeostasis
**At2g02000**	GAD3	glutamate decarboxylase, calmodulin binding	carboxylic acid metabolic process
**At2g02010**	GAD4	glutamate decarboxylase, calmodulin binding	carboxylic acid metabolic process
At2g02490		unknown protein	unknown
At2g02515		unknown protein	unknown
At2g15740		zinc finger (C2H2 type) family protein	regulation of transcription
At2g18490		zinc finger (C2H2 type) family protein	regulation of transcription
**At2g23170**	GH3.3	IAA-amido synthase	response to auxin stimulus/auxin homeostasis
At2g25330		meprin and TRAF homology domain-containing protein	unknown
At2g25700	ASK3	E3 ubiquitin ligase SCF complex subunit SKP1/ASK1 (At3)	(ubiquitin-protein ligase activity)
At2g26050		zinc ion binding	unknown
**At2g30810**		gibberellin-regulated family protein	response to gibberellin stimulus
At2g36560		DNA-binding protein-related	unknown
At2g39640		glycosyl hydrolase family 17 protein	carbohydrate metabolic processes
At2g43670		glycosyl hydrolase family 17 protein	unknown
At2g45110	ATEXPB4	beta-expansin	cellulose and pectin-containing cell wall loosening
At3g02670		proline-rich family protein	phosphate transport
**At3g05460**		sporozoite surface protein-related	unknown
At3g05860	AGL45	MADS-box family transcription factor	regulation of transcription
At3g10780		emp24/gp25L/p24 family protein	intracellular protein transport
			cell redox homeostasis/intracellular signalling
At3g11920		glutaredoxin-related	cascade
At3g17150		pectinesterase inhibitor	(pectinesterase inhibitor activity)
At3g21410	FBW1	F-box family protein	ubiquitin-dependent protein catabolic process
**At3g24510**	GIG2	defensin-like (DEFL) family protein	unknown
At3g49770		unknown protein	unknown
**At3g57160**		unknown protein	unknown
**At3g57270**	BG1	glycosyl hydrolase family 17 protein, beta-1,3-glucanase	carbohydrate metabolic process
At4g16500		cysteine protease inhibitor family protein	(enzyme regulator activity)
At4g25530	FWA	homeodomain protein	regulation of transcription
		small, secreted, cysteine rich protein with sequence similarity to	
At4g29285	LCR24	the PCP (pollen coat protein) gene family	unknown
		chromatin remodeling factor, strong similarity to CHD3	chromatin assembly or disassembly/regulation of
At4g31900		(PICKLE)	transcription
At4g32105		galactosyltransferase	protein amino acid glycosylation
**At4g35725**		unknown protein	unknown
At4g36590	AGL40	MADS-box transcription factor	regulation of transcription
At4g37360	CYP81D2	cytochrome P450 family protein	electron transport
**At4g39650**	GGT2	gamma-glutamyltranspeptidase	glutathione catabolic process
At5g05260	CYP79A2	cytochrome P450	glucosinolate biosynthesitic process
		protease inhibitor/seed storage/lipid transfer protein (LTP)	
**At5g09370**		family protein	lipid transport
At5g09730	BXL3	glycosyl hydrolase family 3 protein, beta-xylosidase	carbohydrate metabolic process
At5g10440	CYCD4;2	cyclin family protein	regulation of progression through cell cycle
At5g12070		self-incompatibility protein-related	unknown
At5g14960	E2L1/DEL2	transcription factor/E2F-like repressor	regulation of progression through cell cycle
At5g20710	BGAL7	beta-galactosidase	lactose catabolic process
At5g34883		unknown protein	unknown
At5g34885		unknown protein	unknown
At5g40040	RPP2E	60S acidic ribosomal protein P2	translational elongation
**At5g46950**		invertase/pectin methylesterase inhibitor family protein	(pectinesterase inhibitor activity)
At5g50480		CCAAT-box binding transcription factor Hap5a	regulation of transcription
At5g54220		defensin-like (DEFL) family protein	Unknown

*bold AtId = also down in 4 × 2, 6 × 2 (SLR/pSLR ≤-0.6)

Up = (SLR/pSLR ≥ 0.6), not up = (SLR/pSLR (≤ 0.3).

GO annotations were obtained from TAIR (http://www.arabidopsis.org/tools/bulk/go/index.jsp; Berardini et al., 2004). GO function terms are provided where no biological process terms were annotated.

#### Genes associated with large/overproliferating seeds

There were 114 genes called up in 2xX4x but not up in crosses generating maternal excess (Additional file 5 table S5, S5.1). More than half (62) were also up in 2xX6x (Table 4), and all but ten of these were likewise overexpressed in *fis1*X2x. This underlines the similarities in transcription profiles not only between interploidy crosses generating different degrees of paternal excess, but between these and *fis1 *mutant seeds. Ninety-five genes were up in 2xX6x and *fis1*X2x but not 4xX2x and 6xX2x (Additional file 5 table S5, S5.2), of which 43 were not also up in 2xX4x, and therefore associated with an extreme paternal excess phenotype. Finally there were 158 genes called down in 4xX2x but not down in 2xX4x and 2xX6x (Additional file 5 table S5, S5.3), including 52 also down in 6xX2x, and 32 down in 4xX2x, 6xX2x, and *msi1*. Twelve genes were up in 2xX4x, 2xX6x, and *fis1*X2x and also down in 4xX2x and 6xX2x (Table 4), indicating especially strong positive association with seed growth.

##### Transcription factors

**Figure 4:**
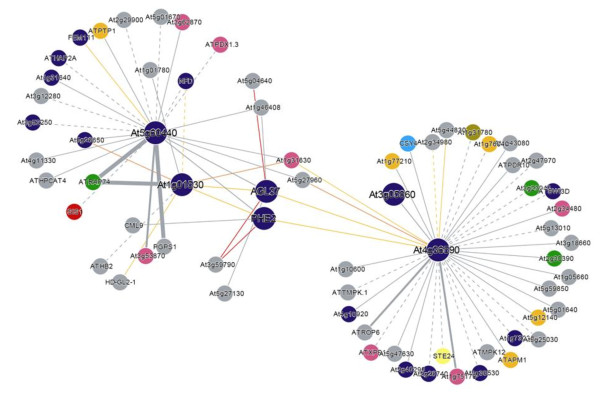
**Interaction map for the AGLs overexpressed in 'big seeds'**. Big blue circles represent the 5 AGLs overexpressed in the 2 × 6 and *fis1*X2x crosses.

MADS-box genes are involved in many aspects of plant development, including essential roles in reproduction such as specification of floral organ identity, and regulatory roles during seed and fruit development [47]. Strikingly, a set of co-regulated and interacting MADS-box transcription factors [48] are associated with large seeds. The Arabidopsis interactome [49] visualized through the interaction viewer http://bbc.botany.utoronto.ca/interactions/ shows the interactions between these MADS box proteins (Figure 4). Similar shared functions of the AGAMOUS-LIKE gene clusters have been demonstrated in early endosperm development and have been implicated in interspecific incompatibility [50,51]. These notably include *PHE1 *(At1g65330) and its close homologue *PHE2 *(At1g65300), which are both overexpressed in 2xX4x, 2xX6x, and *fis1*X2x. *PHE1 *was previously identified as overexpressed in *fis1/mea *mutants in a microarray experiment performed on younger seeds than we used here [28]. *PHE1 *and *PHE2 *share the same Affymetrix probeset and therefore either transcript may have been responsible for the signal. The Agilent array contains separate probes for these two genes but due to sequence similarity, cross-hybridization was still a possibility. However qRT-PCR using gene-specific primers gave similar expression profiles for both genes (Additional file 4 table S4), indicating that both *PHE1 *and *PHE2 *are likely to be upregulated in seeds with paternal excess or a paternalizing *fis1*X2x mutation. This scenario is supported by the observation that *PHE1 *expression is repressed by *FIS1*/*MEA*, an inhibitor of proliferation [10]. In our microarray data, the MADS-box genes *AGL28 *(At1g01530) and *AGL40 *(At4g36950) were called up in one or more of 2xX4x, 2xX6x, or *fis1*X2x, while our qRT-PCR data showed both genes were overexpressed in all three large seed genotypes (Additional file 4 table S4). These MADS-box genes are co-expressed with *PHE1*, and their products interact with the PHE1 and PHE2 proteins in yeast two-hybrid assays [48]. We also tested *AGL62 *(At5g60440) as it was likewise reported to interact with the PHE proteins, and called up in 2xX6x and *fis1*X2x; qRT-PCR showed this gene is upregulated in 2xX4x, 2xX6x, and *fis1*X2x but not up in 4xX2x or 6xX2x. Several seed defects leading to abortion were observed in *agl62 *seeds. These seeds have a low number of endosperm nuclei and exhibit precocious endosperm cellularization as well as embryo defects ([52]; unpublished data Bouariky and Tiwari). A further MADS-box gene called up in all three large seed samples, *AGL45 *(At3g05860), encodes a protein reported to interact with AGL40.

It is interesting to observe that the MADS box proteins interact in different complexes in different backgrounds to regulate endosperm development; AGL 62- AGL80 for endosperm development (Kang et al., 2008), AGL80-AGL61 for central cell development [50] and AGL62-AGL90 endosperm rescue overcoming post zygotic barrier in *A thaliana *× *A arenosa *crosses [51]. Hence the overexpressed AGLs in our dataset may very well interact as higher order complexes to regulate endosperm development in the interploidy crosses and/or be part of a transcriptional cascade promoting seed growth.

Overexpression of individual genes e.g., AGL28, PHE1, AGL40 under the endosperm specific promoter of At5g46950 [53] did not result in any significant change in seed size, overexpression of PHE2 under the same promoter however yielded slightly heavier seeds (unpublished data Tiwari et al). This might indicate that two or more members need to be overexpressed for a visible phenotype. Further experiments such as study of mutants of one or more members of the complex are required to investigate whether upregulation of these MADS-box genes is a cause or a consequence of enhanced seed growth.

*PHE1 *is preferentially expressed in endosperm from the paternal allele only, and therefore its expression trend in our data was as expected. We were surprised however to find an oppositely imprinted gene, *FWA *(At4g25530), also called up in 2xX4x, 2xX6x, and *fis1*X2x. *FWA *is a homeodomain transcription factor that is expressed only in endosperm, and only from the maternal alleles [4,54]. Therefore we expected *FWA *to show a complementary expression trend to *PHE1*, i.e. up in 4xX2x and 6xX2x, but not up in paternal excess crosses. Our qRT-PCR data for *FWA *supports our microarray data for large seeds and additionally calls *FWA *down in 4xX2x, 6xX2x, and *msi1 *(Additional file 4 table S4), indicating the microarry data revealed a *bona fide *expression trend. One possibility to be tested is whether *FWA *expression might be deregulated in a background of parental imbalance, so that it becomes expressed from paternal alleles, or in the embryo, or both. It was recently reported that *PHE1 *loses imprinting in interspecific crosses between *A. thaliana *and *A. arenosa *that also generate paternal excess, becoming ectopically expressed from maternal alleles [55]. Another imprinted gene MPC which is paternally imprinted [12], also shows an upregulation in the 2xX6x, and *fis1*X2x crosses but is unchanged compared to the balanced cross (2xX2x) in the 4xX2x and 6xX2x. It will be interesting to determine whether both FWA and MPC are deregulated in the case of paternal imbalance.

The transcription factor *WRKY10*/*MINISEED3 *(*MINI3*) (At1g55600), up in 2xX6x and *fis1*X2x, also has a known role in seed growth. qRT-PCR confirmed our microarray data and in addition returned an up call for 2xX4x and a down call for 6xX2x (Additional file 4 table S4). This expression trend supports a previously reported role for *MINI3 *in promoting endosperm growth, based on the observation that loss-of-function *mini3 *mutants produce small seeds with small endosperms that cellularize early [56].

##### Genes involved in cell proliferation and chromatin organization

Although paternalized seeds display increased and prolonged endosperm proliferation, and delayed or inhibited cytokinesis, few core cell cycle genes were overexpressed in large seeds. The exceptions included two D cyclins, *CYCD4;1 *(At5g65420) and *CYCD4;2 *(At5g10440). The former was called up in 2xX6x and *fis1*X2x, and the latter was up in all three large seed crosses but not in maternal excess. qRT-PCR confirmed the microarray data for large seeds for both genes, and additionally strengthened the association of *CYCD4;2 *with seed growth by showing it is downregulated in 4xX2x and 6xX2x (Additional file 4 table S4). D cyclins are involved in the G1 to S transition and respond to signals such as cytokinin and sucrose [57]. CYCD4;1 was found to be rate-limiting for cell division in germinating seeds [58]. CYCD4;2 has previously been reported to lack the Rb-binding motif and PEST sequence of other D cyclins, but functional assays show it likewise has a role in proliferation [59]. To our knowledge *CYCD4;2 *is absent or shows extremely low expression in other microarray experiments http://www.genevestigator.ethz.ch and therefore its strong association with seed growth here makes it an interesting candidate for control of cell proliferation in developing seeds. *DEL2/E2Fd/E2L1 *(At5g14960), encoding an atpyical E2F, was called up in 2xX4x and 2xX6x, and down in maternal excess and *msi1*; qRT-PCR indicated a similar trend although comparatively lower upregulation in 2 × 4 (Additional file 4 table S4). Typical E2F proteins heterodimerize with DP proteins to bind E2F sites in promoters of genes associated with DNA synthesis and replication and cell cycle control, including D cyclins, and may be positive or negative regulators of cell division. In contrast, atypical E2Fs bind E2F sites as monomers and repress E2F-regulated promoters [60,61]. The function of DEL2/E2Fd has not yet been described.

Consistent with increased cell proliferation in seeds with paternal excess and inhibited proliferation in maternalized seeds, many genes involved in DNA replication, chromatin organization, RNA translation, or protein synthesis were overexpressed in large seeds and/or down in small seeds. These included histones (*Histone H3*, At1g09200; *Histone H4*, At5g59690), genes involved in chromatin modification (*SUVH7*, At1g17770; *FAS1*, At1g65470; *SDG21/SUVH8*, At2g24740), and members of the Origin of Replication complex (*ATORC2*, At2g37560; *ATORC1a*, At4g14700). SUVH7 and SDG21/SUVH8 are related to animal Su(var)3-9 proteins, which affect chromatin packaging through histone methylation [62], while FAS1 is required for heterochromatin formation [63]. Seeds mutant for *ATORC2 *undergo very few endosperm or embryo divisions before arresting, indicating that this gene is essential for cell proliferation in the seed [64]; downregulation of *ATORC2 *in 4xX2x is consistent with this role. In contrast *ATORC1a *may be involved in endoreduplication rather than proliferation [65]. Although endoreduplication is not a prominent feature of Arabidopsis endosperm as in maize, there is some evidence for endoreduplication in chalazal endosperm [66,67]. *ATORC1a *was called down in both 4xX2x and 6xX2x seeds, and this could be a factor in the suppression of peripheral endosperm cell division or of chalazal endosperm growth observed in seeds with maternal excess.

##### Genes involved in hormone pathways

We observed differential expression of many genes involved in metabolism or signalling of the hormones cytokinin, gibberellin, brassinosteroid, and auxin. According to our microarray data the cytokinin oxidase *CKX2 *(At2g19500) is up in 2xX6x and *fis1*X2x; this was confirmed by qRT-PCR (Additional file 4 table S4), which also indicated overexpression in 2xX4x and *msi1 *and underexpression in 4xX2x crosses. This pattern was at first surprising as cytokinin oxidases catalyse the irreversible degradation of cytokinin, which is associated with cell proliferation. However, recent work shows that *CKX *overexpression increases seed size in *Arabidopsis *to a greater extent than can be completely accounted for by the accompanying loss of fertility [68]. Gibberellic acid (GA) is known to be required for seed germination but there is also evidence that it is essential for seed growth [69]. Several genes involved in GA metabolism or response were upregulated in large seeds: these included *GA1 *(At4g02780), encoding a copalyl diphosphate synthase that catalyses the first committed step in GA biosynthesis, At1g44090, encoding a member of the GA 20-oxidase family that catalyses synthesis of bioactive GA, and At2g30810, a GA-regulated family protein. This last gene had a particularly strong association with seed growth, showing very high levels of overexpression both in microarray data and in qRT-PCR--according to the latter, 18-fold in 2xX4x, 69-fold in 2xX6x, and 30-fold in *fis1*X2x--and severe underexpression in 4xX2x, 6xX2x, and *msi1*. Other microarray experiments show this gene is highly expressed in siliques and also present in isolated seeds http://www.genevestigator.ethz.ch, but no function has been reported. Brassinosteroids (BRs) promote cell growth and division, and are most abundant in pollen and immature seeds [70]. Genes involved in BR synthesis or response that were up in large seeds or down in small seeds included *BSU1 *(At1g03445), encoding a serine threonine phosphatase preferentially expressed in elongating cells which is involved in response to BRs [71]; and *DWF4 *(At3g50660), whose product catalyses a rate-limiting step in BR synthesis [72]. Further genes involved in hormone metabolism and function are annotated on the lists in Additional file 5 table S5, S5.1-3.

#### Genes associated with small/underproliferating seeds

Seeds with maternal excess are characterized by a small seed cavity, inhibited proliferation and early cellularization of endosperm, small chalazal endosperm, and absence of endosperm nodules. One hundred-nineteen genes were overexpressed in 4xX2x but not in 2xX4x and 2xX6x, and of these 31 were also up in 6xX2x, and 16 in *msi1 *as well (Table 5 and Additional file 5 table S5, S5.4). Unfertilized *msi1 *seeds have no chalazal endosperm or nodules, and fewer endosperm nuclei than a fertilized FIS-class mutant would produce, but in contrast to seeds with maternal excess, the seed cavity is not notably small in parthenogenetic *msi1*, endosperm fails to cellularize, and embryo development is very limited. Therefore it is not surprising that there was less overlap between the transcriptional profiles of *msi1 *and seeds with maternal excess than there was between fertilized *fis1 *and seeds with paternal excess. Twelve genes were called down in 2xX4x but not 4xX2x and 6xX2x (Additional file 5 table S5, S5.5), and 14 genes were called down in 2xX6x and *fis1*X2x (Additional file 5 table S5, S5.6).

**Table 5:** Genes called up in 4xX2x and 6xX2x, not up in 2xX4x and 2xX6x.

AtId	symbol	annotation	GO biological process (function)
At1g13080	CYP71B2	cytochrome P450 monooxygenase	heat acclimatino
At1g15100	RHA2A	RING-H2 finger protein	(protein binding)
			response to auxin stimulus
At1g19850	ARF5/MP/IAA24	auxin response factor	(transcription factor activity)
At1g23205		invertase/pectin methylesterase inhibitor family protein	(pectinesterase inhibitor activity)
		DREB subfamily A-5 of ERF/AP2 transcription factor	
At1g46768	RAP2.1	family	regulation of transcription
At1g47960	C/VIF1	cell wall/vacuolar invertase	(pectinesterase inhibitor activity)
At1g56280	ATDI19	drought-responsive family protein	response to water deprivation
At1g70670		caleosin-related family protein	(calcium ion binding)
At1g80170		polygalacturonase/pectinase	carbohydrate metabolic process
At2g03980		GDSL-motif lipase/hydrolase family protein	lipid metabolic process
			auxin polar transport/carotenoid
At2g26170	CYP711A1/MAX1	thromboxane-A synthase	biosynthetic process
At2g37130	PER21	peroxidase	defense response to fungus
At2g37710	RLK	receptor lectin kinase	response to salicylic acid stimulus
At2g39400		hydrolase	aromatic compound metabolic process
At3g11340		UDP-glucoronosyl/UDP-glucosyl transferase family protein	metabolic process
At3g14280		unknown protein	unknown
At3g17790	ATACP5/PAP17	acid phosphatase	cellular phosphate ion homeostasis
At3g62650		binding	transport
At4g10955		lipase class 3 family protein	lipid metabolic process
At4g10960	UGE5	UDP-D-glucose 4-epimerase activity	response to stress
At4g11410		short-chain dehydrogenase/reductase (SDR) family protein	metabolic process
At4g15420		PRLI-interacting factor K	ubiquitin-dependent protein catabolism
At4g18550		lipase class 3 family protein	lipid metabolic process
At4g21590	ENDO3	putative endonuclease	DNA catabolism/stamen development
At4g32180	ATPANK2	pantothenate kinase	coenzyme A biosynthesic process
At5g03200		zinc finger (C3HC4-type RING finger) family protein	N-terminal protein myristoylation
At5g20580		unknown protein	unknown
At5g23660	MTN3	homolog of the Medicago nodulin MTN3	unknown
At5g43810	ZLL/PNH	translation initiation factor	(translation initiation factor activity)
At5g44380		FAD-binding domain-containing protein	response to oxidative stress
At5g46780		VQ motif-containing protein	Unknown

Up = (SLR/pSLR ≥ 0.6), not up = (SLR/pSLR (≤ 0.3).

GO function terms are provided where no biological process terms were annotated.

Few of the genes positively associated with small seeds had an obvious link to their phenotype, suggesting that seed growth is more likely to be restricted by downregulation than overexpression of key genes. Several genes involved in auxin function were associated with small seeds, including the auxin response factor *ARF5/MP *(At1g19850), up in 4xX2x, 6xX2x, and *msi1*, which is involved in embryonic patterning and cell expansion [73], and *CYP711A1/MAX1 *(At2g26170), also up in maternal excess and *msi1. MAX *genes regulate auxin transport capacity by regulating abundance of PIN auxin efflux carrier proteins [74]; it is interesting in this context that *PIN4*, which is upregulated in *max1 *mutants [74,75], is also downregulated in 4xX2x crosses. It was surprising to find *CYCD3;3 *(At3g50070) downregulated in 2xX6x and *fis1*X2x, as this has a similar expression pattern during seed germination to *CYCD4;1 *[58], which is up in these crosses (see above); this could reflect different roles for these D cyclins during earlier seed development.

#### Genes upregulated in FIS-class mutants

Genes overexpressed in both *fis1*X2x and *msi1 *(165 genes, Additional file 5 table S5, S5.7) could include some that are deregulated by FIS-class mutations and involved in the overproliferation of *fis *mutant endosperms. This is supported by the appearance of *PHE1 *in this list, which is controlled by the FIS1- and MSI1-containing PRC2; overexpression of this gene in unfertilized *msi1 *seeds is particularly striking as they contain no paternally contributed alleles, which normally contribute the great majority of *PHE1 *expression. Similarly, ectopic expression of *PHE1 *was reported in unfertilized *fis3/fie *seeds [28]. Further candidate growth-promoting genes overexpressed in *fis1*X2x and *msi1 *are the transcription factors *PHE2*, *AGL45*, and *AGL62*, all discussed above. Other upregulated genes that could be involved in the ectopic seed growth observed in *fis *mutants include the MADS box genes *AGL35 *(At5g26630) and *AGL73 *(At5g38620); *ATGA3OX4 *(At1g80330), encoding a gibberellin 3-oxidase preferentially expressed in flowers and siliques that catalyses synthesis of bioactive GA [76]; and *CPD *(At5g05690), involved in brassinosteroid synthesis.

## Conclusion

**Figure 5:**
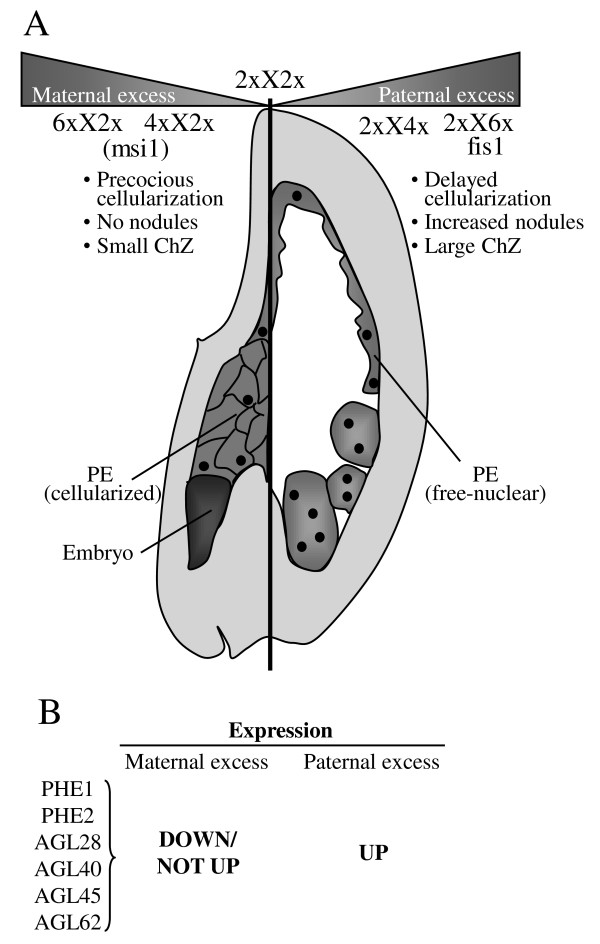
**Relationship between phenotype and gene expression resulting from interploidy crosses and the *fis1 *mutation**. **(A) **Composite seed showing the major phenotypic characteristics of maternal and paternal excess crosses. Cellularization/differentiation of endosperm proceeds from the micropylar ('maternal') to chalazal ('paternal') pole, occurring precociously in maternal excess and late or never in paternal excess. Chz = chalazal endosperm, PE = peripheral endosperm. Transcriptional profiling shows that *fis1*X2x seeds are strongly similar to extreme paternal excess in gene expression as well as phenotype. **(B) **PHE1 and other MADS-box transcription factors that encode interacting proteins are upregulated in paternal excess/*fis1 *and down or unchanged in maternal excess. Therefore these are strong candidates for key regulators of the interploidy cross phenotypes.

Reciprocal interploidy crosses in plants often give complementary seed phenotypes, but little is known about the alterations to transcriptional programmes responsible for this. Here we investigated gene expression underlying the differential development of seeds with paternal or maternal excess. One explanation for interploidy cross phenotypes is that they disrupt the balance of active copies of imprinted genes in the seed. Mutations in FIS-class genes also disrupt imprinting, and fertilized seeds of FIS-class mutants resemble interploidy seeds with lethal paternal excess, while unfertilized FIS-class mutant seeds develop autonomously with no paternal contribution, and have phenotypic attributes of both paternal and maternal excess. Therefore we also profiled fertilized and unfertilized FIS-class mutants to test their transcriptional profiles against seeds with parental genomic imbalance, and to identify genes deregulated by impairment of PRC2 function. Hierarchical clustering and comparison of genes called differentially expressed placed *fis1*X2x seeds in the same group as seeds with paternal excess, showing that the similar phenotypes are indeed underpinned by similar patterns of gene expression. Figure 5A is a schematic diagram of a composite seed summarizing the main features of maternal and paternal excess, and placing the interploidy and *fis1*X2x crosses on the maternal:paternal spectrum based on their transcriptional profiles as described above. *msi1 *autonomous seeds cluster with maternal excess but also show many differences, which may be due to the paternalizing effect of the FIS-class mutation as well as lack of fertilization, and therefore its position on the spectrum is less certain. To learn more about regulation of seed size, we filtered our data for sets of genes strongly associated with enhanced or inhibited seed growth. Genes overexpressed in large seeds but not in small seeds included many candidates for factors controlling seed growth, such as a group of MADS-box transcription factors encoding interacting proteins (Fig. 5B), cell cycle genes, and genes involved in hormone pathways. Some of these genes are also overexpressed in unfertilized FIS-class mutants, suggesting particularly strong association with endosperm proliferation. It would be interesting to study whether any methylation differences that exist between genes in the different interploidy crosses contribute towards the imbalance of imprinted gene expression. Methylation asymmetry has been correlated with the monoallelic expression pattern of imprinted genes in the endosperm and with differences in the expression of genes in the embryo and endosperm in both wildtype as well as *dme *mutant seeds [77,78]. It would also be of interest to look closely at the maternally derived PolIV dependent si-RNAs [79] and the expression pattern of targets of those si-RNAs or progenitors in the interploidy crosses. The work presented here is therefore a step towards understanding the related phenomena of parental genome balance and imprinting.

## Methods

### Plant material and pollinations

The following stocks were used: C24 diploid (2×) hemizygous for an A9-barnase transgene which confers male sterility [80], C24 tetraploid (4×) A9-barnase, Col-1 hexaploid (6×), *fis1-3 *homozygous mutants (= *mea-3*/*f644*, [18]) in the L*er *accession, and *msi1-2 *homozygous mutants [22] in C24. A9-barnase hemizygotes produce male sterile and fertile segregants, which were used as seed and pollen parents respectively in manual pollinations. 6× Col was emasculated two days before pollination to produce the 6xX2x cross. Siliques were harvested at 5 DAP and pooled from at least five plants per cross. For 2xX2x and interploidy crosses, siliques at 5 DAP had a mean length of 1.57 cm ± sem 0.02 with no significant differences among crosses (not shown). *fis1*X2x siliques had somewhat reduced length (1.29 ± 0.04), as L*er *seed parents produce short, blunt siliques.

Homozygous *msi1 *mutants were crossed with 2× C24 A9-barnase, and male sterile plants in the F1 were scored for presence of the *msi1 *mutant allele on the ability of their pistils to elongate without pollination. *msi1 *siliques were harvested at 7 DAF (days after flower opening), which microscopic inspection suggested was comparable to the 5 DAP stage of a balanced cross. *msi1 *rather than *fis1 *mutants were used to generate autonomous seeds as *msi1 *has a much higher penetrance of autonomous endosperm development [21].

### Confocal laser scanning microscopy

Samples were prepared as in Braselton et al. [81] and imaged using an Axiovert 100M Zeiss LSM510 laser scanning microscope. Feulgen-stained samples were excited using an argon ion laser at 488 nm and emissions detected at ≥515 nm. Images measuring 1024 × 1024 pixels were collected using a C-Apochromat 63×/1.2 water lens, saved in TIFF format, and processed using Photoshop version 8.0

### Microarray protocols

#### Affymetrix platform

Total RNA was extracted from whole siliques of 2xX2x (control cross), 2xX4x, 4xX2x, 2xX6x, and 6xX2x (interploidy crosses), and *fis1*X2x crosses at 5 DAP (two biological replicates of each), and unfertilized *msi1 *siliques at 7 DAF, using an RNeasy Plant Mini Kit (Qiagen), concentrated using an RNeasy MinElute Cleanup Kit (Qiagen), and hybridized to Affymetrix ATH1 Genome Arrays http://www.affymetrix.com at the NASC (Nottingham Arabidopsis Stock Centre) microarray facility (affymetrix.arabidopsis.info). An ATH1 array contains 22,746 distinct non-control oligonucleotide probe sets representing approximately 24,000 genes. At the probe level, the arrays were analysed using Affymetrix GCOS (MASv5). For a detailed description of the MASv5 algorithm see Affymetrix' Statistical Algorithms Description Document http://www.affymetrix.com/support/technical/whitepapers/sadd_whitepaper.pdf. A brief summary can be found in Schulz et al. [36]. Using the pairwise comparative variant of the algorithm, each of the eleven experimental samples (two replicates each of four interploidy crosses and *fis1*X2x, and one sample of unfertilized *msi1*) was compared to both biological replicates of the control sample (2xX2x). For each probe set, this analysis yielded a signal log2-ratio (SLR) as a measure of the degree of differential expression between the two samples and a change P-value as a measure of confidence in the expression difference. Each interploidy cross, *fis1*X2x, and unfertilized *msi*1 was compared with both replicates of the control 2xX2x cross, resulting in four SLR values for all experiments with biological replicates and two SLR values for *msi*1.

#### P-value weighting of Affymetrix SLRs

Each Affymetrix probe set is synonymous for ten P-values and SLRs, one for each array comparison (interploidy or *msi1 *versus 2xX2x). To simplify the subsequent analyses, P-value and SLR were combined into a single value pSLR = SLR/(1 + e(2P - .1)/.01)), separately for each array comparison. For P-values close to 0 or 1 that correspond to high statistical confidence in the measured differential expression, the pSLR is essentially identical to the SLR. However, with decreasing statistical confidence, pSLR quickly approaches zero, shrinking large SLRs (both negative and positive) with little supporting statistical evidence for differential expression so that their unreliable and hence, deceptively large values do not interfere with the subsequent analyses. This method has been shown to be a more accurate measure of differential expression than SLR or P-value in isolation [36]. The four pSLR values for each interploidy cross and *fis1*X2x from the four-way comparison were averaged, resulting in a single pSLR for each. The two pSLR values for *msi*1 were also averaged.

#### Agilent platform

mRNA was extracted from whole siliques of 2xX2x and interploidy crosses as above, and *fis1*X2x crosses, using a hot borate method for total RNA extraction [82] and an Oligotex mRNA Midi Kit (Qiagen) to isolate mRNA, and prepared for hybridization to custom Agilent 22K two-dye (Cy3 and Cy5) arrays http://www.chem.agilent.com, each carrying 27,402 non-control 60-mer oligonucleotide probes designed by Ceres, Inc. http://www.ceres-inc.com. For each experimental cross (interploidy or *fis1*X2x), one array was hybridized using a Cy3-labeled sample together with a Cy5-labeled 2xX2x control cross sample using Agilent standard operating protocols. The dyes were then swapped and the experiment repeated on a second array. Each array produced two expression measurements per probe: s_ij _for the control sample and s'_ij _for the experimental sample where i and j identify array and probe, respectively. All subsequent analyses were based on the log2-ratios, log2(s'_ij_/s_ij_), of these values. The ratios from the dye swap experiments were averaged to give a single SLR per probe per cross.

#### Correspondence between Affymetrix probe sets and Ceres probes

The 28,952 cDNA sequences in the ATH1 database (release 5) were searched for exact matches to the 25-mer perfect match (PM) probes on the ATH1 array (277,042 exact PM probe matches). Each Affymetrix probe set contains 11 PM probes. An Affymetrix probe set was considered to match an ATH1 cDNA if 9 out of its 11 PM probes generated exact matches to the cDNA (22,415 probe set matches). The Ceres 60-mer probes were aligned to the ATH1 cDNAs using BLAT (fastMap option) [83]. Only alignments for which Q = number of matches/60 = 5/6 were considered a match, leading to a total of 26,440 matches. Per distinct transcript ('AtId') in the ATH1 database, only the best sense match to the latest version of the transcript was retained, leaving 22,961 matches. In addition, the Ceres probes were directly aligned to the target sequences of the Affymetrix probe sets using BLAT as above, producing an additional 6,983 matches. An Affymetrix probe set and a Ceres probe were then considered to correspond to each other if they matched the same transcript or each other. 20,442 distinct pairs composed of an Affymetrix probe set and a Ceres probe fulfilled this condition.

#### Expression and differential expression pre-filtering

An Affymetrix probe set and its corresponding Ceres probe had to meet the following preconditions on measured absolute and differential expression in order to be included in the subsequent analysis stages. The sum of a Ceres probe's Cy3 and Cy5 signals had to exceed 50 in both dye-swap experiments for at least one cross, which excludes unreliable measurements due to low expression. A similar precondition was applied to Affymetrix probe sets. Specifically, probe sets that were called absent by GCOS (detection P-value > 0.06) on both the balanced cross and all the interploidy and *msi1 *arrays were excluded. These preconditions were met by 15,134 Affymetrix probe sets and their corresponding Ceres probes, which corresponded to 14,944 unique AtIds.

#### Expression change threshold

Data filtering was performed using Microsoft Access. A gene was called up- or down-regulated compared to the balanced 2xX2x cross if it's SLR (Agilent) or pSLR (Affymetrix) was ≥0.6 or ≤-0.6 respectively. This corresponds to a change in expression of approximately 50%. After initial analysis, we only called a gene up or down if these criteria were fulfilled in both platforms. A gene called 'not up' had an SLR and pSLR of ≤ 0.3 (expression change of approximately 20%), and a gene called 'not down' had an SLR and pSLR of ≥ -0.3.

### Hierarchical clustering of samples

Expression data from both Agilent and Affymetrix experiments were loaded into TIGR Mev [84]. A 50% variance filter was applied to the dataset and hierarchical clustering was performed using the Pearson Correlation distance metric and the complete linkage method. To check the robustness of the clusters, clustering of samples was repeated with the Covariance and Cosine correlation distance metrics. Principal Component Analysis of the samples [85] was also done in Mev using the default covariance metric and number of neighbours for KNN imputation value of 10.

### qRT-PCR

Crosses were performed and siliques harvested as for microarray experiments (above). Total RNA was extracted from whole siliques using an RNeasy Mini Kit (Qiagen) and subjected to an on-column DNase treatment and a further in-solution DNase treatment. Extracted RNA was further purified and concentrated using an RNeasy MinElute Cleanup Kit (Qiagen) following manufacturer's instructions. Total RNA was quantified using a spectrophotometer (WPA Lightwave) and integrity checked by gel electrophoresis. Primers were designed and checked for secondary structures, hairpins and dimers using NetPrimer (Premier Biosoft International). A standard RT-PCR was done using the primers, and amplification of a single product and absence of primer dimers was verified by standard gel electrophoresis.

qRT-PCR was performed using a Superscript III Platinum Two Step qRT-PCR Kit (Invitrogen). Briefly 1 μg of total RNA was used along with oligo dt and random primers and first strand cDNA synthesis was done at 46°C for 90 min. The synthesized cDNA was treated with RNaseH. Different dilutions of the cDNA (1:10, 1:20, 1:30, and 1:50) were prepared and 2 μl of each dilution were used in a PCR reaction. Primers and annealing temperatures are shown in Additional file 6 table 6 online.

Four genes were tested for suitability as controls for constant expression across all the samples. These were At1g13440 (*GAPDH*), At3g04120 (*GAPC*), At3g18780 (*Actin2*), and At5g44200 (*CBP20*). Of these *GAPC *was found to have the most consistent expression and was used as a control for normalizing differential transcription efficiency and RNA amounts. Real-time PCR was done on a DNA Engine Opticon 2 (Bio-Rad). Typical cycling conditions were 95°C for 8 min followed by 45 cycles of 95°C for 15 sec, annealing temp X (see Additional file 6 table 6) for 15 sec, 72°C for 20 sec; plate read after each cycle; melting curve 38-98°C read every 0.5°C hold for 6 sec. Opticon 2 software calculates the reaction efficiencies without the need of a standard curve (Opticon 2 manual). The efficiencies were used to calculate the relative expression levels of the candidate genes in the experimental samples (siliques from interploidy crosses, *fis1*x2x or unpollinated *msi1*) compared to the control sample (2xX2x) at 5 DAP using the pffafl equation [86] where Ratio = (Efficiency Target)^ΔCT target (2xX2x-experimental sample)^/(Efficiency Ref)^ΔCT ref (2xX2x-experimental sample)^. Amplification of a single product was verified by a melting curve at the end of the reaction. Two to nine reactions were run per sample, depending on the variability observed, and averaged to obtain a mean expression level.

### Accession numbers

Agilent and Affymetrix microarray data have been desposited in the GEO public repository http://www.ncbi.nlm.nih.gov/projects/geo/ under the accession numbers GSE20007.

## Authors' contributions

ST carried out all the RNA extractions, prepared samples for Affymetrix microarray work, and all of the qRT PCR work. She also performed the downstream data analysis as well as helped prepare the manuscript. RS has processed the raw microarray data and done the normalisations and the statistical calculations. MS and RJS have prepared the manuscript as well as helped design the experimental programme and gave intellectual guidance. RP and group (AS, KZ) have performed the Agilent microarray experiment and provided the raw data. RO and GK have given intellectual inputs to the programme. All authors have read and approved the final manuscript.

## Supplementary Material

Additional file 1 **Masterlist containing unique Atids used for further analysis**. **Table S1**. List of Genes called present in at least one seed sample along with corresponding probesets, and averaged SLRs and pSLRs for each platform (Ag = Agilent; Af = Affymetrix). **Table S1.1**. List of Early seed specific genes (Day et.al. 2008) present in the dataset. Click here for file

Additional file 2 **Upregulated and downregulated genes in individual platforms**. **Table S2**. List of Genes called up (Affymetrix pSLR or Agilent SLR ≥ 0.6) or down (≤ -0.6) in each sample in the individual platforms. Click here for file

Additional file 3 **Upregulated and downregulated genes common in both platforms**. **S3**. List of genes that are upregulated or downregulated in both the platforms. Click here for file

Additional file 4 **qRT-PCR results**. **S4**. qRT-PCR data files and assembled graphs depicting changes to gene expression of selected genes and their agreement with both the Agilent and Affymetrix microarray data. Click here for file

Additional file 5 **Genes associated with paternal excess, maternal excess, or FIS-class mutations**. **S5**. Analysis of all the interploidy crosses and the *fis *mutants to find genes associated with paternal excess, maternal excess, or FIS-class mutations. **Table S5.1**. 2xX4x up (4xX2x, 6xX2x not up). Genes also up in 2xX6x and *fis1*X2x are marked. **Table S5.2**. 2xX6x, *fis1*X2x up (4xX2x, 6xX2x not up). Genes also up in 2xX4x are marked. **Table S5.3 **4xX2x down (2xX4x, 2xX6x not down). Genes also down in 6xX2x and *msi1 *are marked. **Table S5.4 **4xX2x up (2xX4x, 2xX6x not up). Genes also up in 6xX2x and *msi1 *are marked. **Table S5.5 **2xX4x down (4xX2x, 6xX2x not down). **Table S5.6 **2xX6x, *fis1*X2x down (4xX2x, 6xX2x not down) **Table S5.7 ***fis1*X2x, msi1 up. Click here for file

Additional file 6 **Additional information about the qRT-PCR**. **S6**. Primers and annealing temperatures for qRT-PCR. Click here for file

## References

[B1] Haig D Westoby M Genomic imprinting in endosperm: its effect on seed development in crosses between species, and between different ploidies of the same species, and its implications for the evolution of apomixis Phil Trans R Soc Lond B 1991 333 1 13 10.1098/rstb.1991.0057

[B2] Gehring M Choi Y Fischer RL Imprinting and seed development Plant Cell 2004 16 S203 S213 10.1105/tpc.017988 15010515 PMC2643396

[B3] Kinoshita T Yadegari R Harada JJ Goldberg RB Fischer RL Imprinting of the *MEDEA *Polycomb gene in the *Arabidopsis *endosperm Plant Cell 1999 11 1945 1952 10.1105/tpc.11.10.1945 10521524 PMC144115

[B4] Kinoshita T Miura A Choi Y Kinoshita Y Cao X Jacobsen SE Fischer RL Kakutani T One-way control of *FWA *imprinting in *Arabidopsis *endosperm by DNA methylation Science 2004 303 521 523 10.1126/science.1089835 14631047

[B5] Vielle-Calzada J-P Thomas J Spillane C Coluccio A Hoeppner MA Grossniklaus U Maintenance of genomic imprinting at the *Arabidopsis MEDEA *locus requires zygotic DDM1 activity Genes and Development 1999 13 2971 2982 10.1101/gad.13.22.2971 10580004 PMC317158

[B6] Guo M Rupe MA Danilevskaya ON Yang X Hu Z Genome-wide mRNA profiling reveals heterochronic allelic variation and a new imprinted gene in hybrid maize endosperm Plant Journal 2003 36 30 44 10.1046/j.1365-313X.2003.01852.x 12974809

[B7] Gutiérrez-Marcos JF Pennington PD Costa LM Dickinson HG Imprinting in the endosperm: a possible role in preventing wide hybridization Philosophical Transactions of the Royal Society of London B 2003 358 1105 1111 10.1098/rstb.2003.1292 PMC1693205 12831476

[B8] Gutiérrez-Marcos JF Costa LM Biderre-Petit C Khbaya B O'Sullivan DM Wormald M Perez P Dickinson HG *maternally expressed gene1 *is a novel maize endosperm transfer cell-specific gene with a maternal parent-of-origin pattern of expression Plant Cell 2004 16 1288 1301 10.1105/tpc.019778 15105441 PMC423216

[B9] Gutiérrez-Marcos JF Costa LM Dal Prà M Scholten S Kranz E Perez P Dickinson HG Epigenetic asymmetry of imprinted genes in plant gametes Nature Genetics 2006 38 876 878 10.1038/ng1828 16823380

[B10] Köhler C Page DR Gagliardini V Grossniklaus U The *Arabidopsis thaliana *MEDEA *Polycomb *group protein controls expression of *PHERES1 *by parental imprinting Nature Genetics 2005 37 28 30 15619622 10.1038/ng1495

[B11] Jullien PE Kinoshita T Ohad N Berger F Maintenance of DNA methylation during the *Arabidopsis *life cycle is essential for parental imprinting Plant Cell 2006 18 1360 1372 10.1105/tpc.106.041178 16648367 PMC1475502

[B12] Tiwari S Schulz R Ikeda Y Dytham L Bravo J Mathers L Spielman M Guzmán P Oakey RJ Kinoshita T Scott RJ *MATERNALLY EXPRESSED PAB C-TERMINAL*, a novel imprinted gene in Arabidopsis, encodes the conserved C-terminal domain of polyadenylate binding proteins Plant Cell 2008 20 2387 2398 10.1105/tpc.108.061929 18796636 PMC2570725

[B13] Jahnke S Scholten S Epigenetic resetting of a gene imprinted in plant embryos Current Biology 2009 19 1677 1681 10.1016/j.cub.2009.08.053 19781944

[B14] Scott RJ Spielman M Bailey J Dickinson HG Parent-of-origin effects on seed development in *Arabidopsis thaliana* Development 1998 125 3329 3341 9693137 10.1242/dev.125.17.3329

[B15] Olsen O-A Nuclear endosperm development in cereals and *Arabidopsis thaliana* Plant Cell 2004 16 S214 S227 10.1105/tpc.017111 15010513 PMC2643391

[B16] Cooper DC Caryopsis development following matings between diploid and tetraploid strains of Zea mays American Journal of Botany 1951 38 702 708 10.2307/2437917

[B17] Leblanc O Pointe C Hernandez M Cell cycle progression during endosperm development in *Zea mays *depends on parental dosage effects Plant Journal 2002 32 1057 1066 10.1046/j.1365-313X.2002.01491.x 12492846

[B18] Kiyosue T Ohad N Yadegari R Hannon M Dinneny J Wells D Katz A Margossian L Harada JJ Goldberg RB Fischer RL Control of fertilization-independent endosperm development by the *MEDEA *polycomb gene in *Arabidopsis* Proc Natl Acad Sci USA 1999 96 4186 4191 10.1073/pnas.96.7.4186 10097185 PMC22442

[B19] Vinkenoog R Spielman M Adams S Fischer RL Dickinson HG Scott RJ Hypomethylation promotes autonomous endosperm development and rescues post-fertilisation lethality in *fie *-mutants Plant Cell 2000 12 2271 2282 10.1105/tpc.12.11.2271 11090224 PMC150173

[B20] Sorensen MB Chaudhury AM Robert H Bancharel E Berger F Polycomb group genes control pattern formation in plant seed Current Biology 2001 11 277 281 10.1016/S0960-9822(01)00072-0 11250158

[B21] Köhler C Hennig L Bouveret R Gheyselinck J Grossniklaus U Gruissem W Arabidopsis MSI1 is a component of the MEA/FIE Polycomb group complex and required for seed development EMBO J 2003 22 4804 4814 10.1093/emboj/cdg444 12970192 PMC212713

[B22] Guitton A-E Page DR Chambrier P Lionnet C Faure JE Grossniklaus U Berger F Identification of new members Fertilisation Independent Seed Polycomb Group pathway involved in the control of seed development in *Arabidopsis thaliana* Development 2004 131 2971 2981 10.1242/dev.01168 15151989

[B23] Spielman M Vinkenoog R Dickinson HG Scott RJ The epigenetic basis of gender in flowering plants and mammals Trends in Genetics 2001 17 705 711 10.1016/S0168-9525(01)02519-7 11718924

[B24] Chanvivattana Y Bishopp A Schubert D Stock C Moon YH Sung ZR Goodrich J Interaction of Polycomb-group proteins controlling flowering in *Arabidopsis* Development 2004 131 5263 5276 10.1242/dev.01400 15456723

[B25] Guitton A-E Berger F Control of reproduction by Polycomb Group complexes in animals and plants Int J Dev Biol 2005 49 707 716 10.1387/ijdb.051990ag 16096976

[B26] Jullien PE Katz A Oliva M Ohad N Berger F Polycomb group complexes self-regulate imprinting of the Polycomb group gene *MEDEA *in Arabidopsis Current Biology 2006 16 486 492 10.1016/j.cub.2006.01.020 16527743

[B27] Makarevich G Leroy O Akinci U Schubert D Clarenz O Goodrich J Grossniklaus U Köhler C Different Polycomb group complexes regulate common target genes in Arabidopsis EMBO Rep 2006 7 947 952 10.1038/sj.embor.7400760 16878125 PMC1559666

[B28] Köhler C Hennig L Spillane C Pien S Gruissem W Grossniklaus U The Polycomb-group protein MEDEA regulates seed development by controlling expression of the MADS box gene *PHERES1* Genes Dev 2003 17 1540 1553 10.1101/gad.257403 12815071 PMC196083

[B29] Ohad N Margossian L Hsu YC Williams C Repetti P Fischer RL A mutation that allows endosperm development without fertilization Proc Natl Acad Sci USA 1996 93 5319 5324 10.1073/pnas.93.11.5319 11607683 PMC39243

[B30] Chaudhury AM Ming L Miller C Craig S Dennis ES Peacock W Fertilization-independent seed development in *Arabidopsis thaliana* Proc Natl Acad Sci USA 1997 94 4223 4228 10.1073/pnas.94.8.4223 9108133 PMC20611

[B31] Guitton A-E Berger F Loss of function of MULTICOPY SUPPRESSOR OF IRA 1 produces nonviable parthenogenetic embryos in *Arabidopsis* Current Biology 2005 15 1 6 10.1016/j.cub.2005.02.066 15854908

[B32] Pylatuik JD Fobert PR Comparison of transcript profiling on *Arabidopsis *microarray platform technologies Plant Molecular Biology 2005 58 609 624 10.1007/s11103-005-6506-3 16158238

[B33] Grossniklaus U Vielle-Calzada J-P Hoeppner MA Gagliano W Maternal control of embryogenesis by *MEDEA*, a Polycomb group gene in *Arabidopsis* Science 1998 280 446 450 10.1126/science.280.5362.446 9545225

[B34] Swarbreck D Wilks C Lamesch P Berardini TZ Garcia-Hernandez M Foerster H Li D Meyer T Muller R Ploetz L Radenbaugh A Singh S Swing V Tissier C Zhang P Huala E The *Arabidopsis *Information Resource (TAIR): gene structure and function annotation Nucleic Acids Research 2008 36 D1009 D1014 10.1093/nar/gkm965 17986450 PMC2238962

[B35] Day RC Herridge RP Ambrose BA Macknight RC Transcriptome analysis of proliferating Arabidopsis endosperm reveals biological implications for the control of syncytial division, cytokinin signalling, and gene expression regulation Plant Physiology 2008 148 1964 1984 10.1104/pp.108.128108 18923020 PMC2593665

[B36] Schulz R Menheniott TR Woodfine K Wood AJ Choi JD Oakey RJ Chromosome-wide identification of novel imprinted genes using microarrays and uniparental disomies Nucleic Acids Research 2006 34 Art No. e88 10.1093/nar/gkl461 PMC1524921 16855283

[B37] Guo A He K Liu D Bai S Gu X Wei L Luo J DATF: a database of *Arabidopsis *transcription factors Bioinformatics 2005 21 2568 2569 10.1093/bioinformatics/bti334 15731212

[B38] Skibbe DS Wang X Zhao X Borsuk LA Nettleton D Schnable PS Scanning microarrays at multiple intensities enhances discovery of differentially expressed genes Bioinformatics 2006 22 1863 1870 10.1093/bioinformatics/btl270 16731695

[B39] Eisen MB Spellman PT Brown PO Botstein D Cluster analysis and display of genome-wide expression patterns Proc Natl Acad Sci USA 1998 95 14863 14868 10.1073/pnas.95.25.14863 9843981 PMC24541

[B40] Berardini TZ Mundodi S Reiser R Huala E Garcia-Hernandez M Zhang P Mueller LM Yoon J Doyle A Lander G Moseyko N Yoo D Xu I Zoeckler B Montoya M Miller N Weems D Rhee SY Functional annotation of the Arabidopsis genome using controlled vocabularies Plant Physiol 2004 135 1 11 10.1104/pp.104.040071 PMC514112 15173566

[B41] Beissbarth T Speed TP GOstat: find statistically overrepresented Gene Ontologies within a group of genes Bioinformatics 2004 20 1464 1465 10.1093/bioinformatics/bth088 14962934

[B42] Erilova A Brownfield L Exner V Rosa M Twell D Scheid OM Hennig L Köhler C Imprinting of the Polycomb group gene *MEDEA *serves as a ploidy sensor in Arabidopsis PLoS Genet 2009 5 9 e1000663 10.1371/journal.pgen.1000663 19779546 PMC2738949

[B43] Menges M de Jager SM Gruissem W Murray JAH Global analysis of the core cell cycle regulators of Arabidopsis identifies novel genes, reveals multiple and highly specific profiles of expression and provides a coherent model for plant cell cycle control Plant Journal 2005 41 546 566 10.1111/j.1365-313X.2004.02319.x 15686519

[B44] Lauber MH Waizenegger I Steinmann T Schwarz H Mayer U Hwang I Lukowitz W Jürgens G The *Arabidopsis *KNOLLE protein is a cytokinesis-specific syntaxin J Cell Biol 1997 139 1485 1493 10.1083/jcb.139.6.1485 9396754 PMC2132613

[B45] Strompen G El Kasmi F Richter S Lukowitz W Assaad FF Jürgens G Mayer U The *Arabidopsis HINKEL *gene encodes a kinesin-related protein involved in cytokinesis and is expressed in a cell cycle-dependent manner Current Biology 2002 12 153 158 10.1016/S0960-9822(01)00655-8 11818068

[B46] Vanstraelen M Inzé D Geelen D Mitosis-specific kinesins in *Arabidopsis* Trends in Plant Science 2006 11 167 175 10.1016/j.tplants.2006.02.004 16530461

[B47] Becker A Theissen G The major clades of MADS-box genes and their role in the development and evolution of flowering plants Molecular phylogenetics and evolution 2003 29 464 489 10.1016/S1055-7903(03)00207-0 14615187

[B48] de Folter S Immink RGH Kieffer M Paøenicová L Henz SR Weigel D Busscher M Kooiker M Colombo L Kater MM Davies B Angenent GC Comprehensive interaction map of the Arabidopsis MADS box transcription factors Plant Cell 2005 17 1424 1433 10.1105/tpc.105.031831 15805477 PMC1091765

[B49] Lee JG O'Toole N Ammar R Provart NJ Millar AH Geisler M A predicted interactome for Arabidopsis Plant Physiology 2007 145 317 329 10.1104/pp.107.103465 17675552 PMC2048726

[B50] Steffen JG Kang I Portereiko MF Lloyd A Drews GN AGL61 interacts with AGL80 and is required for central cell development in Arabidopsis Plant Physiology 2008 148 59 268 10.1104/pp.108.119404 PMC2528130 18599653

[B51] Walia H Josefsson C Dilkes B Kirkbride R Harada J Comai L Dosage-dependent deregulation of an AGAMOUS-LIKE gene cluster contributes to interspecific incompatibility Current Biology 2009 19 13 1128 1132 10.1016/j.cub.2009.05.068 19559614 PMC6754343

[B52] Kang IH Steffen JG Portereiko MF Lloyd A Drews GN The AGL62 MADS domain protein regulates cellularization during endosperm development in Arabidopsis The Plant Cell 2008 20 635 647 10.1105/tpc.107.055137 18334668 PMC2329934

[B53] Tiwari S Spielman M Day RC Scott RJ Proliferative phase endosperm promoters from *Arabidopsis thaliana* Plant Biotechnology Journal 2006 4 393 407 10.1111/j.1467-7652.2006.00189.x 17177805

[B54] Soppe WJ Jacobsen SE Alonso-Blanco C Jackson JP Kakutani T Koornneef M Peeters AJ The late flowering phenotype of fwa mutants is caused by gain-of-function epigenetic alleles of a homeodomain gene Mol Cell 2000 6 791 802 10.1016/S1097-2765(05)00090-0 11090618

[B55] Josefsson C Dilkes B Comai L Parent-dependent loss of gene silencing during interspecies hybridization Current Biology 2006 16 1322 1328 10.1016/j.cub.2006.05.045 16824920

[B56] Luo M Dennis ES Berger F Peacock WJ Chaudhury A *MINISEED3 *(*MINI3*), a *WRKY *family gene, and *HAIKU2 *(*IKU2*), a leucine-rich repeat (*LRR*) *KINASE *gene, are regulators of seed size in *Arabidopsis* Proc Natl Acad Sci USA 2005 102 17531 17536 10.1073/pnas.0508418102 16293693 PMC1297679

[B57] Oakenfull EA Riou-Khamlichi C Murray JAH Plant D-type cyclins and the control of G1 progression Phil Trans R Soc Lond 2002 357 749 760 10.1098/rstb.2002.1085 PMC1692988 12079670

[B58] Masubelele NH Dewitte W Menges M Maughan S Collins C Huntley R Nieuwland J Scofield S Murray JAH D-type cyclins activate division in the root apex to promote seed germination in Arabidopsis Proc Natl Acad Sci USA 2005 102 15694 15699 10.1073/pnas.0507581102 16227434 PMC1266134

[B59] Kono A Ohno R Umeda-Hara C Uchimiya H Umeda M A distinct type of cyclin D, *CYCD4;2*, involved in the activation of cell division in *Arabidopsis* Plant Cell Rep 2006 25 540 545 10.1007/s00299-005-0075-4 16408177

[B60] Kosugi S Ohashi Y E2Ls, E2F-like repressors of *Arabidopsis *that bind to E2F sites in a monomeric form J Biol Chem 2002 277 16553 16558 10.1074/jbc.M200913200 11867638

[B61] Mariconti L Pellegrini B Cantoni R Stevens R Bergounioux C Cella R Albani D The E2F family of transcription factors from *Arabidopsis thaliana* J Biol Chem 2002 277 9911 9919 10.1074/jbc.M110616200 11786543

[B62] Baumbusch LO Thorstensen T Krauss V Fischer A Naumann K Assalkhou R Schulz I Reuter G Aalen RB The *Arabidopsis thaliana *genome contains at least 29 active genes encoding SET domain proteins that can be assigned to four evolutionarily conserved classes Nucleic Acids Research 2001 29 4319 4333 10.1093/nar/29.21.4319 11691919 PMC60187

[B63] Schönrock N Exner V Probst A Gruissem W Hennig L Functional genomic analaysis of CAF-1 mutants in *Arabidopsis thaliana* Journal of Biological Chemistry 2006 281 9560 9568 10.1074/jbc.M513426200 16452472

[B64] Collinge MA Spillane C Köhler C Gheyselinck J Grossniklaus U Genetic interaction of an origin replication complex subunit and the *Polycomb *Group gene *MEDEA *during seed development Plant Cell 2004 16 1035 1046 10.1105/tpc.019059 15020747 PMC412875

[B65] Diaz-Trivino S del Mar Castellano M de la Paz Sanchez M Ramirez-Parra E Desvoyes B Gutierrez C The genes encoding *Arabidopsis *ORC subunits are E2F targets and the two *ORC1 *genes are differently expressed in proliferating and endoreduplicating cells Nucleic Acids Research 2005 33 5404 5414 10.1093/nar/gki854 16179646 PMC1236721

[B66] Boisnard-Lorig C Colon-Carmona A Bauch M Hodge S Doerner P Bancharel E Dumas C Haseloff J Berger F Dynamic analyses of the expression of the HISTONE::YFP fusion protein in Arabidopsis show that syncytial endosperm is divided in mitotic domains Plant Cell 2001 13 495 509 10.1105/tpc.13.3.495 11251092 PMC135513

[B67] Baroux C Fransz P Grossniklaus U Nuclear fusions contribute to polyploidization of the gigantic nuclei in the chalazal endosperm of Arabidopsis Planta 2004 220 38 46 10.1007/s00425-004-1326-2 15248065

[B68] Werner T Köllmer I Bartrina I Holst K Schmülling T New insights into the biology of cytokinin degradation Plant Biology 2006 8 371 381 10.1055/s-2006-923928 16807830

[B69] Swain SM Singh DP Tall tales from sly dwarves: novel functions of gibberellins in plant development Trends in Plant Science 2005 10 123 129 15749470 10.1016/j.tplants.2005.01.007

[B70] Shimada Y Goda H Nakamura A Takatsuto S Fujioka S Yoshida S Organ-specific expression of brassinosteroid-biosynthetic genes and distribution of endogenous brassinosteroids in Arabidopsis Plant Physiology 2003 131 287 297 10.1104/pp.013029 12529536 PMC166808

[B71] Mora-García S Vert G Yin Y Caño-Delgado A Cheong H Chory J Nuclear protein phosphatases with Kelch-repeat domains modulate the response to brassinosteroids in *Arabidopsis* Genes and Development 2004 18 448 460 10.1101/gad.1174204 14977918 PMC359398

[B72] Choe S Dilkes BP Fujioka S Takatsuto S Sakurai A Feldmann KA The *DWF4 *gene of Arabidopsis encodes a cytochrome P450 that mediates multiple 22α-hydroxylation steps in brassinosteroid biosynthesis Plant Cell 1998 10 231 244 10.1105/tpc.10.2.231 9490746 PMC143988

[B73] Hardtke CS Ckurshumova W Vidaurre DP Singh SA Stamatiou G Tiwari SB Hagen G Guilfoyle TJ Berleth T Overlapping and non-redundant functions of the *Arabidopsis *auxin response factors *MONOPTEROS *and *NONPHOTOTROPIC HYPOCOTYL 4* Development 2004 131 1089 1100 10.1242/dev.00925 14973283

[B74] Bennett T Sieberer T Willett B Booker J Luschnig C Leyser O The *Arabidopsis MAX *pathway controls shoot branching by regulating auxin transport Current Biology 2006 16 553 563 10.1016/j.cub.2006.01.058 16546078

[B75] Lazar G Goodman HM MAX1, a regulator of the flavonoid pathway, controls vegetative axillary bud outgrowth in Arabidopsis Proc Natl Acad Sci USA 2006 103 472 476 10.1073/pnas.0509463102 16387852 PMC1324789

[B76] Mitchum MG Yamaguchi S Hanada A Kuwahara A Yoshioka Y Kato T Tabata S Kamiya Y Sun T-P Distinct and overlapping roles of two gibberellin 3-oxidases in Arabidopsis development Plant Journal 2006 45 804 818 10.1111/j.1365-313X.2005.02642.x 16460513

[B77] Gehring M Bubb KL Henikoff S Extensive demethylation of repetitive elements during seed development underlies gene imprinting Science 2009 324 1447 1451 10.1126/science.1171609 19520961 PMC2886585

[B78] Hsieh TF Ibarra CA Silva P Zemach A Eshed-Williams L Fischer RL Zilberman D Genome-wide demethylation of Arabidopsis endosperm Science 2009 324 1451 1454 10.1126/science.1172417 19520962 PMC4044190

[B79] Mosher RA Melnyk CW Kelly KA Dunn RM Studholme DJ andBaulcombe DC Uniparental expression of PolIV-dependent siRNAs in developing endosperm of Arabidopsis Nature 2009 460 283 286 10.1038/nature08084 19494814

[B80] Paul W Hodge R Smartt S Draper J Scott R The isolation and characterisation of the tapetum-specific *Arabidopsis thaliana *A9 gene Plant Mol Biol 1992 19 611 622 10.1007/BF00026787 1627774

[B81] Braselton JP Wilkinson MJ Clulow SA Feulgen staining of intact plant tissues for confocal microscopy Biotech Histochem 1996 71 55 102 10.3109/10520299609117139 9138536

[B82] Wilkins TA Smart LB Krieg P Isolation of RNA from plant tissue A laboratory guide to RNA: isolation, analysis and synthesis 1996 New York: Wiley-Liss 21 41

[B83] Kent JW BLAT - the BLAST-like alignment tool Genome Research 2002 12 656 664 11932250 10.1101/gr.229202 PMC187518

[B84] Saeed AI Sharov V White J Li J Liang W Bhagabati N Braisted J Klapa M Currier T Thiagarajan M Sturn A Snuffin M Rezantsev A Popov D Ryltsov A Kostukovich E Borisovsky I Liu Z Vinsavich A Trush V Quackenbush J TM4: a free, open-source system for microarray data management and analysis Biotechniques 2003 34 374 378 12613259 10.2144/03342mt01

[B85] Raychaudhuri S Stuart JM Altman RB Principal components analysis to summarize microarray experiments: application to sporulation time series Pacific Symposium on Biocomputing 2000 2000 452 463 10.1142/9789814447331_0043 PMC2669932 10902193

[B86] Pfaffl MW A new mathematical model for relative quantification in real-time RT-PCR Nucleic Acids Research 2001 29 2002 2007 10.1093/nar/29.9.e45 PMC55695 11328886

